# Foliar Application of an Amino Acid-Enriched Urea Fertilizer on ‘Greco’ Grapevines at Full Veraison Increases Berry Yeast-Assimilable Nitrogen Content

**DOI:** 10.3390/plants9050619

**Published:** 2020-05-13

**Authors:** Alessandro Mataffo, Pasquale Scognamiglio, Antonio Dente, Daniela Strollo, Giuseppe Colla, Youssef Rouphael, Boris Basile

**Affiliations:** 1 Department of Agricultural Sciences, University of Naples Federico II, 80055 Portici, Italy; alessandro.mataffo@unina.it (A.M.); pasquale.scognamiglio2@unina.it (P.S.); youssef.rouphael@unina.it (Y.R.); 2 Mastroberardino Winery, 83042 Avellino, Italy; dente@mastroberardino.com (A.D.); strollo@mastroberardino.com (D.S.); 3 Department of Agriculture and Forest Sciences, University of Tuscia, 01100 Viterbo, Italy; giucolla@unitus.it

**Keywords:** biostimulant, YAN, leaf application, *Vitis vinifera*, *Saccharomyces cerevisiae*, fermentation, ammonium, aminic, nitrogenous maturity

## Abstract

Reaching a sufficient yeast assimilable nitrogen (YAN) content in berries at harvest is considered a main viticultural goal for wine-making, because low YANs can slow down must fermentation and have negative effects on wine sensory attributes. For this reason, many attempts have been made to define correct fertilization strategies to stimulate YAN accumulation in the berries. Foliar application of amino acid-enriched urea fertilizer is considered a promising environmentally friendly strategy for improving the yield and nutrient efficiency of plants. The aim of this two-year research was to study the effects of two fertilizers based on urea enriched with amino acids applied at low doses in diverse phenological stages on berry YAN concentration in ‘Greco’ grapevines. The results of this study indicate that amino acid-enriched urea fertilizers induced an increase in YANs in the ‘Greco’ berries at harvest, but only when the application was undertaken at full veraison. Foliar applications applied at veraison onset or post-veraison appeared to be ineffective. In addition, the fertilizers enhanced YAN accumulation in the berry without modifying the other composition parameters measured in this study (total soluble solids, titratable acidity, pH and malic acid). Therefore, the results of our study suggest that foliar application of urea fertilizers enriched with amino acids is an effective strategy to increase yeast-assimilable nitrogen concentration in grapevine berries at harvest.

## 1. Introduction

Yeast assimilable nitrogen (YAN) content is a key factor in the fermentation kinetics of musts [1]. Indeed, an insufficient must YAN concentration was reported to slow down fermentation and to cause the undesired presence of thiols and higher alcohols in the wine [1,2] with detrimental alteration of the wine aromatic profile (i.e., formation of unpleasant off-flavors) [3]. To solve such deficiency, nitrogen (N) is usually added to the must, but this kind of supplement represents a significant cost for the wineries and affects their operational logistics. Many genetic (cultivar, rootstock, clone) environmental (climate, soil) and vineyard management (soil, canopy and irrigation managements, harvest time) factors can cause an unsatisfactory YAN concentration in the berries at harvest [1,4]. Among these, the most important appears to be the genetic material (cultivar and the rootstock) [5,6], the pedoclimatic characteristics [7], the environmental conditions [8] and the vineyard agricultural management practices [9]. Soil application of N was reported to effectively increase YANs in grapevine berries [10,11], but this strategy can be expensive with serious economic and environmentally negative consequences [12]. Since the pollution related to N fertilization represents a main environmental concern in many cropping systems around the world [13], research into efficient and environmentally friendly fertilization strategies represents the main goal of many growers, extension specialists, scientists as well as in agricultural governmental policies [14,15,16]. Foliar application of N is generally considered more environmentally friendly compared to soil application because it directly targets the plant [17,18,19] reducing the risk of nitrate leaching [20]. Furthermore, foliar plant nutrition is considered very versatile because it provides the possibility to take prompt action even when early signs of nutrient deficiency are detected [18]. Canoura et al. [21] reported that foliar N fertilization is more effective in increasing berry YAN concentration compared to soil drench fertilization, but the effectiveness of foliar fertilization depends on the timing of application [22]. In the context of a more sustainable agriculture, an interesting strategy to improve nutrient uptake, efficiency and crop performance is represented by the use of molecules with biostimulant action [23]. In particular, protein hydrolysates (PH) which are a mixture of peptides and free amino acids, are considered a useful tool to improve plant performance and N status with a low impact on the environment [24]. This makes the PHs virtually a suitable solution to improve nitrogen-use efficiency in those conditions where the traditional N application is limited by environmental constraints (presence of superficial aquifers, nitrate vulnerable areas, risk of imbalance in soil C/N ratio, etc.). In addition to this positive effect on vine nutritional status, the application of PHs can also represent an effective tool in activating secondary metabolite pathways such as the phenolic biosynthesis in the berries [25], boosting the quality characteristics of grape and wine composition. Research has investigated the suitability of using PHs and/or amino acid-based products as a foliar nutrition to increase YAN concentration in the must, but the literature is not always consistent in this respect [26,27,28,29]. However, the beneficial effects of PHs may depend on several interactive parameters such as protein source (animal or plant origin) or method of protein hydrolysis (chemical or enzymatic) that affect their specific amino acid composition [27,28,29]. In addition, the specific phenological stage when the PHs are applied may also significantly influence the effectiveness of the treatment, and to the best of our knowledge nothing is known about the optimal phenological stage when applying PHs in grapevines. Different authors studied the possibility to enhance the effectiveness of inorganic nitrogen fertilizers by adding amino acids as enhancer [27]. Gutiérrez-Gamboa et al. [30] reported that the foliar application of a urea fertilizer added with amino acids can significantly increase YAN concentration in the berries of ‘Cabernet Sauvignon’ at harvest compared to vines fertilized only with urea and to unfertilized plants. The aim of this two-year experiment was to analyze the effect of two fertilizers based on urea enriched with amino acids applied at low doses in different specific phenological stages on berry YAN concentration in ‘Greco’ grapevines. The cultivar ‘Greco’ was selected because it is one of the most important Italian white cultivars for the production of premium wines and because for this variety an adequate YAN concentration in berry juice at harvest (at least 200 mg N/L) [1] is a necessary prerequisite for reaching high qualitative standards of the wines.

## 2. Results

### 2.1. Climatic Data

**Figure 1:**
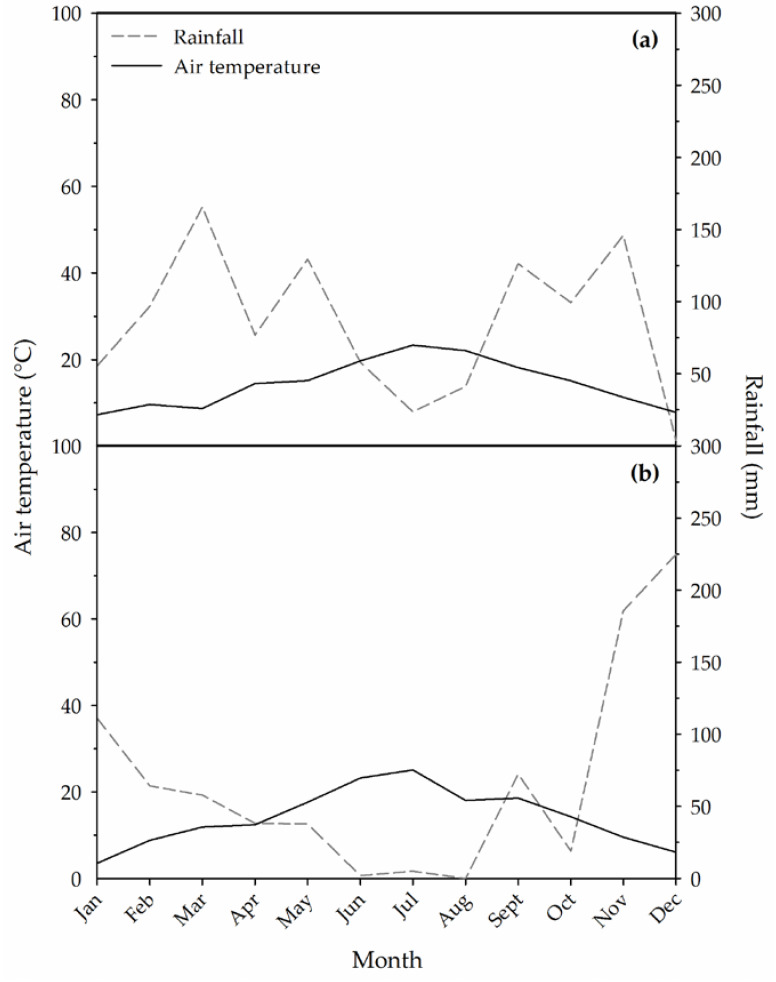
Walter climate diagram reporting monthly mean air temperatures and total rainfall recorded in 2016 (**a**) and 2017 (**b**).

**Figure 2:**
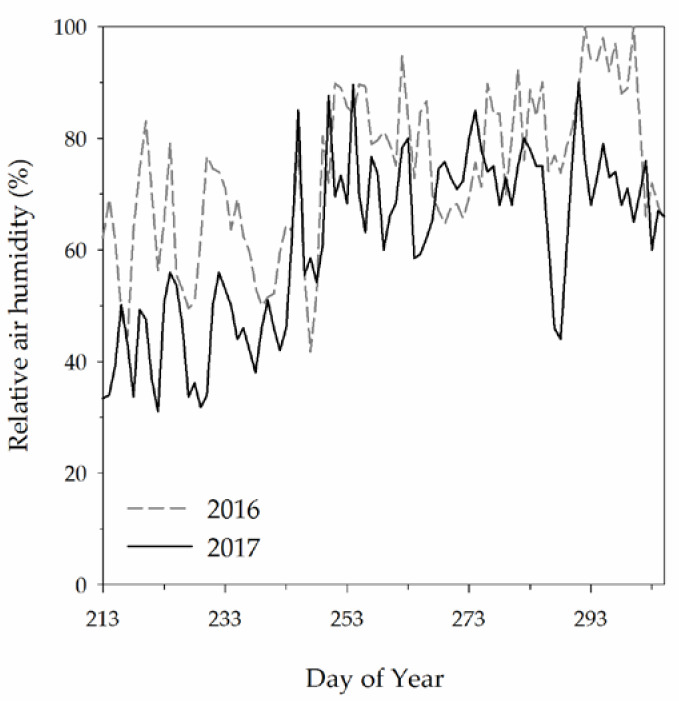
Seasonal pattern of daily mean relative air humidity recorded between 1 August and 30 October in 2016 and 2017.

The first year of the experiment was cooler than 2017 (Figure 1). The average growing season temperature was 18.9 and 20.2 °C in 2016 and 2017, respectively. Thus, the two seasons could be classified as “warm” and “hot” respectively [31]. The Winkler index (calculated from 1 April to 31 October) ranged between 1768.5 and 1992.5 growing degree days (GDD) in 2016 and 2017 respectively. According to this index, the climate of the growing area can be assigned to Region III or Region IV. In 2017, relative air humidity was lower throughout the growing season compared to 2016 (on average 71 and 58% in 2016 and 2017, respectively). Total rainfall from 1 April to 5 October (harvest date) was 555 and 155 mm, respectively, in 2016 and 2017, with almost no rain during August 2017. As suggested by the Walter climate diagrams (Figure 1), the second year of the experiment was characterized by a more intense and longer dry period compared to 2016. Relative air humidity tended to be slightly higher in 2016 than in 2017 throughout the three summer months (Figure 2).

### 2.2. Yeast Assimilable Nitrogen (YAN) Concentration

**Figure 3:**
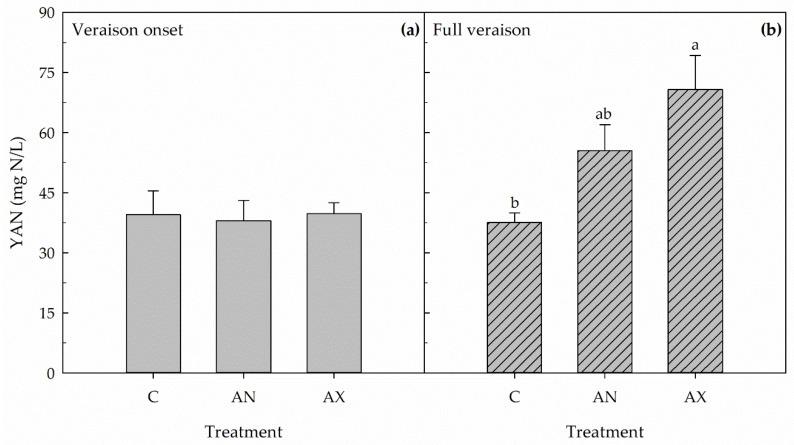
Yeast assimilable nitrogen (YAN) concentration in berries measured at harvest in 2016 in control untreated plants (C) and in vines sprayed at veraison onset (**a**) or full veraison (**b**) with Aminoprotein (AN) or Aminomax (AX). Within each panel, means (n = 8) followed by different letters are significantly different according to Duncan’s test (*p* ≤ 0.05).

**Figure 4:**
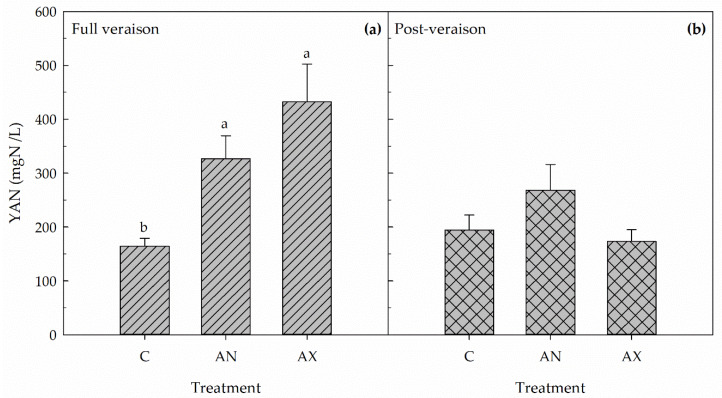
Yeast assimilable nitrogen (YAN) concentration in berries measured at harvest in 2017 in control untreated plants (C) and in vines sprayed at full veraison (**a**) or post-veraison (**b**) with Aminoprotein (AN) or Aminomax (AX). Within each panel, means (n = 8) followed by different letters are significantly different according to Duncan’s test (*p* ≤ 0.05).

In both years, the foliar fertilization (F), the phenological stage (PS), and the F × PS interaction significantly affected YAN concentration in the berries at harvest (Table 1; Figure 3 and Figure 4). In 2016, the foliar application of Aminomax-N or Aminoprotein (hereafter called AX and AN, respectively) applied at veraison onset did not affect YAN concentration in the berries at harvest compared to untreated control vines (Figure 3a), whereas AX application at full veraison induced a significant increase in berry YAN concentration (+89%) compared to control vines (Figure 3b). Indeed, vines treated at full veraison with AN had intermediate berry YAN concentration at harvest compared to the other two treatments (Figure 3b). In 2017, foliar applications at full veraison of both AN and AX induced a significant increase in berry YAN concentration (+99% and +163%, respectively) compared to control (Figure 4a), whereas no effect on this parameter was measured when the two fertilizers were applied in post-veraison (Figure 4b). Independently of the treatment, YAN concentration in the berry juice at harvest was much higher in 2017 compared to 2016.

### 2.3. Aminic and Ammonium N

In 2016, F, PS and F × PS interaction significantly affected aminic N concentration, whereas these factors did not affect ammonium N concentration (Table 1 and Table 2). In this year, AX applied at full veraison induced a more than two-fold increase in aminic N concentration (+117%) compared to control vines (Table 2) whereas aminic N concentration was intermediate in AN vines. In 2017, F, PS and F × PS interaction significantly affected ammonium N concentration, whereas aminic N concentration was not affected (Table 1 and Table 2). Indeed, AX applied at full veraison induced a more than three-fold increase in ammonium N concentration (+237%) compared to control vines (Table 2) whereas ammonium N concentration was intermediate in AN vines. Differences between treatments in both aminic and ammonium N concentration were not significant when the fertilizers were applied at veraison onset or post-veraison (Table 2).

### 2.4. Total Soluble Solids (TSS), pH, Malic Acid, and Total Acidity

In both years, F, PS and F × PS interaction did not affect TSS, titratable acidity, pH and malic acid concentration in the berry juice (Table 1). In 2017, titratable acidity and malic acid concentration were lower than in 2016.

## 3. Discussion

Foliar application of fertilizers based on urea enriched with amino acids induced a significant increase of YAN concentration in the berries of ‘Greco’ grapevines, but the effectiveness of these products were dependent on the phenological stage of application. Indeed, the treatment applied at full veraison consistently increased YAN in both years, whereas the two fertilizers appeared to be ineffective when applied at veraison onset or post-veraison. These results suggest that the ureic/amino acidic N applied at full veraison is more easily absorbed by leaves and/or is more efficiently transported to the berries compared to applications at veraison onset and post-veraison. Foliar nutrient uptake is regulated by leaf permeability that depends on the ultrastructure and the composition of leaf cuticle [32] and is affected by genetic, developmental and environmental factors [17]. Previous studies have reported that in different cultivated plant species leaf permeability to urea can decrease with leaf age because of a progressive increase in cuticle thickness and in wax accumulation [33,34]. Cuticle wax was reported to accumulate progressively during leaf aging also in grapevines [35]. Even though grapevine canopies at any specific phenological stage are a collection of leaves of different ages, in rain-fed vineyards in Mediterranean climates average canopy age is expected to increase from veraison onset to harvest because, in this phenological stage, new vegetative growth is suppressed by summer high air temperature, water stress and competition with berry growth. Scognamiglio [36] reported that in a vineyard adjacent to that used for our trial, vegetative growth of ‘Greco’ grapevines was already over by the end of July. Therefore, it is possible to hypothesize that in our study the lower effectiveness of foliar N nutrition applied late in the vegetative season (post-veraison) was due to an age-related decrease in leaf permeability to the applied nutrients [37,38]. Previous studies on grapevines reported that the N assimilation by leaves was low when urea was applied late in the growing season [22]. Leaf cuticle permeability to nutrients can be also increased by high air humidity. However, this environmental parameter appears to account only partially for the differences in the effectiveness of fertilizer application applied at the three phenological stages. Indeed, in 2016 relative air humidity was slightly higher at full veraison than at veraison onset (79% and 71%, respectively) and this may account for part of the differences in the fertilizer effectiveness between these timings of application. However, this was not the case for 2017 when relative air humidity was lower at full veraison than in post-veraison (61% and 70%, respectively). In addition, foliar nutrition at full veraison was effective in both vegetative seasons independently of the difference between years in relative air humidity. Another possible hypothesis to explain the variability in berry composition induced by the phenological stage of fertilizer application can be related to differences in nitrogen translocation from leaves to berries. Grapevine fruits are sink organs for N and their demand for this nutrient changes during fruit development [10]. In details, in ‘Cabernet Sauvignon’ grapevines, most of the N was reported to be accumulated in the berry in the middle part of fruit ripening (TSS between 14 and 20 °Brix), whereas relatively low accumulation rates were found during the first and the last parts of berry ripening (up to 14 °Brix and after 20 °Brix, respectively). Similarly, Garde-Cerdán et al. [4] reported that berries of ‘Grenache’ grapevines had maximum N demand in the middle part of ripening. In addition, Lasa et al. [22] reported that, in ‘Merlot’, nitrogen translocation from leaves to berries was maximum when urea (labelled with 15N) was applied to leaves during veraison compared to earlier or later spraying times (pre- and post-veraison, respectively). Our results appear to be consistent with this physiological framework of grapevines.

In our study, the AX application was more effective than AN in increasing YAN in the berries at harvest. This occurred despite the fact that the total amount of N applied with the AN treatment (three sprayings) was almost 2.2 times higher than that supplied with AX (1.92 and 0.86 kg N/ha, respectively). Canoura et al. [21] reported that, in ‘Chardonnay’ grapevines, soil and foliar N application (ammonium nitrate and urea, respectively) at fruit-set and veraison stimulated YAN accumulation in the berries and this effect was stronger when the amount of N supplied was doubled (from 32.5 to 65.0 kg N/ha). Similarly, Jiménez-Moreno et al. [39] also reported, for ‘Tempranillo’ grapevines, that the effect of foliar urea application on YAN concentration in the berries increased with the dose of N applied per hectare (0, 2 and 4 kg N/ha). The main difference between the two fertilizers used in our study was the fraction of amino acid included in the formulation, that was higher in AX than in AN (39% and 7.5% of the total N applied, respectively). Amino acids can enhance the assimilation of urea inducing a better N absorption rate and an increase in YAN in berries of Cabernet Sauvignon grapevines [30]. It is well established that PHs can enhance nutrient assimilation processes through upregulation of genes encoding for enzymes involved in the assimilation of inorganic nutrients [40,41,42,43,44]. Similarly, Schiavon et al. [45] reported that an alfalfa-derived PH promoted nitrogen assimilation in plants via a coordinated regulation of C and N metabolism.

Despite the fact that the stimulation of YAN accumulation in the berries induced by both fertilizers applied at full veraison was consistent in both years, the N components of berry YAN that was affected changed depending on the season. Indeed, in 2016 fertilizers induced a significant increase in aminic N concentration but no effect was measured in the ammonium N concentration, whereas in 2017 the effect was opposite compared to the previous year with significant differences only in ammonium N concentration. Nitrogen and carbon metabolisms are strongly interlinked and are strongly affected by weather conditions [46,47]. Any environmental condition causing significant carbon starvation in the vines (high temperature, water stress, etc.) has been reported to impair amino acid synthesis and, therefore, to induce an accumulation of inorganic N in plant organs [46,47]. In 2017, the climate conditions during berry ripening were very hot and dry (compared to 2016; Figure 1) and we observed that ‘Greco’ vines experienced moderate-to-severe symptoms of water stress (yellowing and senescence of basal leaves, etc.). In addition, berry malic-acid concentration at harvest was very low in 2017 compared to 2016 and this also indicates that in 2017 berries were exposed to high air temperatures during ripening. Indeed, high berry temperature induces an increase in cell respiration rates, causing malic acid degradation [48,49]. Finally, our data cannot fully exclude the hypothesis that the fertilizers can also be effective at veraison onset and in post-veraison when specific weather conditions occur at these phenological stages (conditions that are different from those that characterized our trial in 2016 and 2017).

## 4. Materials and Methods 

### 4.1. Experimental Site and Plant Material

The trial was conducted in 2016 and 2017 in a rain-fed commercial vineyard located in Montefusco, Avellino, Italy (41°01′54’’ N, 14°51′55’’ E). According to the USDA Soil Taxonomy [50], the soil was classified as Aquandic Endoaqualf with a clay-loam surface Ap horizon (34.4% sand, 32.6% silt, 33.0% clay) and a clay sub-surface Bt horizon (18.2% sand, 30.4% silt, 51.4% clay). The other physical and chemical properties of the Ap and Bt horizons were, respectively, the following: (a) 8.6 and 8.8 of pH; (b) 0.41 and 0.48 dS m^−1^ of electrical conductivity; (c) 12.2 and 4.5 g kg^−1^ of organic carbon; (d) 9 and 6 g kg^−1^ of calcium carbonate (CaCO_3_); (e) 0.11 and 0.13 g kg^−1^ of total nitrogen (Kjeldhal method); (f) 8.9 and 36 g kg^−1^ of available phosphorus; (g) 26.2 and 27.8 cmol kg^−1^ of exchangeable calcium; (h) 1.9 and 2.3 cmol kg^−1^ of exchangeable potassium; (i) 4.3 and 8.6 cmol kg^−1^ of exchangeable magnesium. The vineyard was planted in the 2007 with ‘Greco’ (*Vitis vinifera* L.) [51] grapevines grafted onto 420A (*Vitis berlandieri* × *V. riparia*) rootstocks and had a slope of 15% facing south. Vines were trained to a unilateral Guyot leaving, with dormant pruning, one horizontal 1-year-old cane bearing 10 buds and one 2-bud spur per plant. Canopies were trained according to a vertical shoot positioning. Vine spacing was 2.50 m × 1.00 m (4000 vines/ha) and rows had a north-south orientation. The vineyard was managed according to the protocol for wine production defined by the “Greco di Tufo DOCG” Denomination of Origin [52]. Under vine weed control was done mechanically on a 80 cm-wide soil strip (40 cm on each side of the vine rows). Fertilization was undertaken by applying, after harvest, 120 kg/ha of organic soil amendments (cow and horse manure; Stalfert N2; Organazoto Fertilizzanti S.p.A.; San Miniato, Pisa, Italy).

### 4.2. Experimental Design and Treatments

The experimental design was a randomized block design with six treatments and four blocks (10 vines/treatment/block) with two untreated rows between the blocks (used as borders). The six treatments consisted of two types of fertilizers applied at two phenological stages and two controls sprayed only with water on the same dates when fertilizers were applied. The products tested in this study were two N fertilizers based on urea enriched with amino acids (Aminomax-N and Aminoprotein, AX and AN, respectively; Meristem, Valencia, Spain). The amino acids included in both products were obtained by enzymatic hydrolysis of plant-derived proteins. AX was characterized by 9% total N content (3.5%, 5.2% and 0.3% of organic, ureic and ammonium N, respectively), whereas AN had 20% total N content (1.5%, 17.0% and 1.5% of organic, ureic and ammonium N, respectively). For each spraying, the application dose per hectare was 3.2 L of product diluted in 800 L of water, corresponding to an application of 288 and 640 g N/ha for AX and AN, respectively. The products were uniformly distributed on the canopies using a backpack sprayer. The two fertilizers were applied at 10-day intervals starting at different specific phenological stages. In 2016, these phenological stages were: veraison onset (10% of yellow berries which occurred on 26 August 2016 corresponding to 1350 GDD accumulated from 1 January and soluble solids content in the berry juice of 10 °Brix) and full veraison (90% of yellow berries that occurred on 6 September 2016 corresponding to 1484 GDD accumulated from 1 January and soluble solids content in the berry juice of 17 °Brix). In 2017 the phenological stages were: full veraison (90% of yellow berries, that occurred on 27 August 2017 corresponding to 1575 GDD accumulated from 1 January and soluble solids content in the berry juice of 18 °Brix) and post-veraison (when soluble solids content in the berry juice reached 21 °Brix, that occurred on 10 September 2017 corresponding to 1721 GDD accumulated from 1 January).

### 4.3. Grape Composition at Harvest

Grapes were harvested manually when the berries of control vines reached a total soluble solids content (TSS) of 23 °Brix. Therefore, to determine harvest date, berry TSS accumulation was monitored weekly starting in the first week of September, around a month before the expected harvest date. This occurred on 4 and 5 October in 2016 and 2017, respectively. At harvest, two 100-berry samples were randomly collected for each treatment and block (8 samples per treatment representing 8 biological replicates per treatment). Each berry sample was separately hand-crushed and the berry juice obtained was used for measuring TSS, pH, titratable acidity (TA) and the concentrations of NH4^+^, aminic N, and malic acid. Berry TSS was measured with a digital refractometer (HI96811. Hanna Instruments, Carrollton, TX, USA). Ten mL of filtered juice was used for pH and TA measurements. The TA was measured by titrating the filtered juice with a 1 N NaOH up to the end point of pH 7.0. Juice pH was measured using a digital pH (GLP 21, Crison, Alella, Barcelona, Spain). In addition, a berry juice sample of 10 mL was centrifuged for four minutes at 2379 × g and five mL of supernatant were used to measure NH4^+^, aminic N, and malic acid concentration using a multi-analyzer Miura one (Tecnología Difusión Ibérica, Barcelona, Spain) as previously described by González-Santamaría et al. [53]. YAN concentration was calculated adding NH4^+^ and aminic N concentrations.

### 4.4. Statistical Analysis

The significance of the effects of the foliar fertilization (F), the phenological stage (PS), and the F × PS interaction on the measured berry composition parameters was assessed with two-way analysis of variance (ANOVA) using Duncan’s post hoc test (P < 0.05) for mean separation. Before the ANOVAs were carried out, the homogeneity of the variance was tested with Levene’s test, and the presence of outliers was checked using the interquartile range method (with 1.5 as constant). All the statistical analyses were performed with the statistical software package SPSS (IBM, Chicago, IL, USA). 

## 5. Conclusions

Foliar application of urea fertilizers enriched with amino acids appears to be an effective strategy to increase yeast-assimilable nitrogen concentration in grapevine berries. This may help oenologists in either decreasing the amount of N added to the must or in avoiding the need for this supplementation. The results of our study suggest that the identification of the right phenological stage for their application seems to be the major factor affecting the effectiveness of this practice. For ‘Greco’ grapevines, this sensitive phenological stage appears to be full veraison, despite the large differences in the weather conditions between the two years of the experiment. In addition, our results suggest that the phenological window when berry YAN accumulation is responsive to these fertilizers is quite narrow. Therefore, a successful use of these products may require a careful monitoring of vine phenology by viticulturists. The relative amount of amino acids included in the formulation appears also to play a role in determining the effectiveness in enhancing urea fertilizers applied at relatively low doses.

## Figures and Tables

**Table 1:** Marginal mean for yeast assimilable N (YAN), ammonium N (NH4+-N), aminic N, total soluble solids (TSS), pH, titratable acidity (TA), and malic acid of berry juice in control vines (C) and vines treated with Aminomax (AX) and Aminoprotein (AN) in two different phenological stages in 2016 and 2017. The significance of the effect of fertilizer (F), phenological stage of application (PS), and F × PS interaction, measured with two-way analysis of variance is also reported. Marginal means followed by different letters are significantly different according to the Duncan’s test (*p* ≤ 0.05). The experimental design considered, for each measured parameter, a total of 8 biological replications (n = 8).

	YAN (mg N/L)	NH_4_^+^-N (mg N/L)	Aminic N (mg N/L)	TSS (°Brix)	pH	TA (g/L Tartaric Acid)	Malic Acid (g/L)
First year							
*Fertilizer (F)*							
C	38.5b	18.0	20.5b	23.3	2.70	10.1	2.38
AX	55.2a	19.5	35.7a	23.1	2.75	9.4	2.29
AN	46.7ab	17.0	29.7a	22.8	2.74	9.5	2.30
*Phenological stage (PS)*							
Veraison onset	39.1b	15.3	23.7b	23.2	2.73	9.7	2.42
Full veraison	54.6a	21.0	33.6a	23.0	2.73	9.7	2.22
*Probability ^Z^*							
F	*	ns	**	ns	ns	ns	ns
PS	**	ns	**	ns	ns	ns	ns
F × PS	*	ns	*	ns	ns	ns	ns
Second Year							
*Fertilizer (F)*							
C	179.5b	112.2b	67.2	22.9	3.07	7.3	0.54
AX	302.7a	230.4a	72.4	22.6	3.12	6.8	0.56
AN	297.6a	224.0a	73.6	22.5	3.08	7.3	0.58
*Phenological stage (PS)*							
Full veraison	307.8a	307.8a	234.7	73.1	3.09	7.1	0.54
Post-veraison	212.1b	212.1b	143.0	69.1	3.09	7.1	0.59
*Probability*							
F	*	*	ns	ns	ns	ns	ns
PS	*	*	ns	ns	ns	ns	ns
F × PS	**	*	ns	ns	ns	ns	ns

^Z^ * = *p* ≤ 0.05; ** = *p* ≤ 0.01; ns = not significant.

**Table 2:** Ammonium nitrogen (NH_4_^+^N) and aminic nitrogen (mean ± standard error of the mean) measured at harvest in 2016 and 2017 in control untreated plants (C) and in vines sprayed at veraison onset, full veraison or post-veraison with Aminoprotein (AN) or Aminomax (AX). Within columns and separated by year, means (n = 8) followed by different letters are significantly different according to Duncan’s test (*p* ≤ 0.05).

Year	Treatment	Veraison Onset		Full Veraison		Post-Veraison	
		NH_4_^+^-N (mg N/L)	Aminic N (mg N/L)	NH_4_^+^-N (mg N/L)	Aminic N (mg N/L)	NH_4_^+^-N (mg N/L)	Aminic N (mg N/L)
2016	C	19.5 ± 4.4	20.0 ± 1.7	16.5 ± 2.2	21.0 ± 1.8b	-	-
	AX	13.7 ± 2.4	26.0 ± 0.9	25.2 ± 3.9	45.5 ± 6.5a	-	-
	AN	12.7 ± 2.7	25.2 ± 2.5	21.2 ± 5.9	34.2 ± 2.9ab	-	-
2017	C	-	-	104.2 ± 7.8b	60.2 ± 7.0	120.2 ± 11.3	74.2 ± 16.6
	AX	-	-	351.0 ± 71.7a	81.2 ± 5.9	109.7 ± 12.5	63.5 ± 9.5
	AN	-	-	249.0 ± 41.9ab	77.7 ± 7.4	199.0 ± 42.5	69.5 ± 8.9
